# Clinical and biochemical characterization of high risk and not high risk for cardiovascular disease adults in a population from peripheral region of Bangladesh

**DOI:** 10.1186/s12889-015-1919-7

**Published:** 2015-06-18

**Authors:** Kaniz Fatema, Nicholas Arnold Zwar, Zebunnesa Zeba, Abul Hasnat Milton, Bayzidur Rahman, Liaquat Ali

**Affiliations:** Department of Epidemiology, Bangladesh University of Health Sciences (BUHS), 125/1, Darus Salam, Mirpur, Dhaka, 1216 Bangladesh; The School of Public Health and Community Medicine, Faculty of Medicine, The University of New South Wales, Sydney, NSW 2052 Australia; Department of Biochemistry, BUHS, 125/1 Darus Salam, Mirpur, Dhaka, 1216 Bangladesh; Centre for Clinical Epidemiology and Biostatistics (CCEB), The School of Medicine and Public Health, Faculty of Health, The University of Newcastle, Newcastle, NSW 2008 Australia; Department of Molecular and Cell Biology, BUHS, 125/1 Darus Salam, Mirpur, Dhaka, 1216 Bangladesh

**Keywords:** Prevalence, Cardiovascular disease, Hypertension, Diabetes, Risk factors, Obesity, Bangladesh

## Abstract

**Background:**

A group of 63708 Bangladeshi adults from a rural area were screened in 2011–12 for cardiovascular diseases (CVD) risk using a questionnaire based tool developed as part of the ‘WHO CVD-RISK Management Package for low-and medium resource setting’. In the current study participants who were found to be high risk and a sample of the not high risk participants from the screening were further characterized clinically and biochemically to explore the burden and determinants of CVD risk factors in a remote rural Bangladeshi population.

**Methods:**

The high risk participants comprised all 1170 subjects who screened positive in 2011–12 and the not high risk group comprised 563 randomly sampled participants from the 62538 who screened negative. Socio-demographic, behavioral, anthropometric, clinical and biochemical data (glucose and lipids) were collected by standardized procedures. Body Mass Index (BMI) was classified following Asian BMI criteria. Data was analyzed using univariable and multivariable methods.

**Results:**

On univariable analysis in high risk and not high risk participants respectively, age in years (M ± SD) was 50 ± 11 for both groups, ratio of male: female was 40:60 and 66:44, current smoking 28.5 % and 50.6 %; smokeless tobacco use 37.1 % and 34.8 %; overweight and obesity measured by body mass index (BMI) was 39.1 % and 20.5 %; high waist circumference (WC) 36.1 % and 11.9 %; high waist to hip ratio (WHR) 53.8 % and 26.3 %; and with high waist to height ratio (WHtR) 56.4 % and 28.4 %, existence of hypertension (HTN) was 15.8 % and 3.6 %, pre-HTN 43.8 % and 12.1 %, diabetes (DM) 14.0 % and 10.5 %, pre-DM 16.9 % and 12.1 % and dyslipidaemia 85.8 % and 89.5 %. In multivariable logistic regression analysis female sex, BMI, WC, WHR and WHtR, HTN and dyslipidaemia remain significantly more common among high risk participants (p < 0.05 and p < 0.001).

**Conclusions:**

The prevalence of clinical and biochemical risk factors of CVDs are quite high even in this rural population and this may be related to the socioeconomic and cultural transition in Bangladeshi society. Surprisingly more of the high risk group was female and there were fewer smokers. Obesity and hypertension were more frequent in high risk participants.

## Background

Cardiovascular diseases (CVDs) are now the major cause of death worldwide. It is estimated by World Health Organization (WHO) that 17.3 million people (31 % of all deaths) died from CVDs in 2012. Approximately 80 % of deaths occurred in low- and middle-income countries (LMICs), predominately in people aged > 60 year [1]. Bangladesh is a LMIC where the emerging challenge in the health sector is non-communicable diseases (NCDs) and, among all NCDs, the foremost cause of death and disability is CVDs [1, 2]. As a whole CVDs and their known risk factors account for 13.4 % of disability adjusted life years (DALYs) lost in Bangladesh [3]. The number of people who are 60 years and above is expected to increase dramatically to 40.5 million by 2050 which would constitute 19 % of the total population of Bangladesh [4, 5]. If early prevention and long-term management measures are not adopted, this growing older population may suffer from multiple conditions due to various CVDs and therefore place a major burden on the Bangladeshi health system [3, 5].

Estimation of CVD risk is central for rational management and prevention of these disorders as well as for designing long-term policies and programs to combat the challenge. It is now known that many of the risk factors of CVDs like smoking, hypertension, dyslipidaemia, diabetes, physical inactivity and obesity are potentially modifiable by health counselling [6]. However, it is also known that the prevalence of these risk factors may vary substantially from population to population and even in various subgroups of the same population. Understanding of these variations is essential to design appropriate evidence based strategies to face the emerging problem.

To address this gap in knowledge a group of agreed 63708 Bangladeshi adults, living in a peripherally located area of Bangladesh, was screened (in 2011–12) using a WHO recommended tool, designed to estimate CVD risk in low resource settings [7]. The aim of this study was to describe and contrast the prevalence of CVD risk factors in the high risk and a sample of the not high risk members of the cohort. We also examined the association between socio-demographic, behavioral, anthropometric and clinical measures between these two groups of study participants. This study is expected to add to knowledge about the prevalence of established and emerging CVD risk factors in the Bangladeshi population.

## Methods

**Fig. 1:**
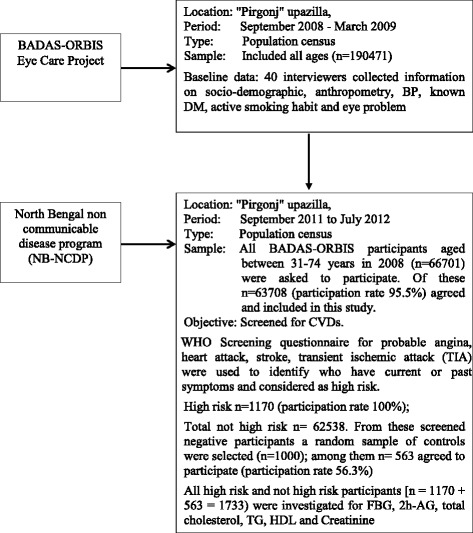
Flow chart for the study design and selection of participants

The original cohort was initiated in 2008 under the ‘BADAS-ORBIS Eye Care Project’. The project was designed to generate epidemiological data on the burden of diabetic retinopathy and associated risk factors from a rural population and study area (approx. 300 km from the capital) of Thakurgaon district of Bangladesh. The cohort had total n = 66701 participants who were aged between 31–74 years in 2008. In 2011–12, a screening program was run using a questionnaire based tool under the North Bengal Non-Communicable Disease Program (NB-NCDP) of Bangladesh University of Health Sciences (BUHS). The tool was developed as part of the ‘WHO CVD-RISK Management Package for low- and medium – resource settings’ and following the recommendations of the WHO [7]. The tool consists of a total of eight questions to screen for probable angina, heart attack, stroke, transient ischemic attack (TIA). People who have pre-existing CVDs are known to be at high risk of experiencing another cardiovascular event [8]. It does not include measurement of any biochemical as well as a number of important clinical risk factors. Of 63708 BADAS-ORBIS participants who agreed to take part in NB-NCDP (95.5 % participation rate), 1733 were found to be at high risk for CVDs as assessed by the tool (paper submitted for publication). Detail screening methodology has been described in Fig. 1.

The current study was planned basing on our chain hospital [(i.e., Thakurgaon Swasthaseba Hospital TSH)] under the Health Care Development Project (HCDP) of Bangladesh Diabetes Somity (BADAS). To ensure uniformity in recruitment of participants and data collection, a team of 20 data collectors were given two weeks of intensive training on study protocol, questionnaire administration, techniques of examination [i.e., electrocardiography (ECG)] and evaluation. All interviewers were selected based on their competency, at least 12 years of education completed, and prior experience in conducting interviews, surveys and using the census method.

Among the 1733 participants of the present study, 1170 comprised of all the members of a high risk group identified by the 2011–12 survey using the WHO recommended 2002 tool. The remaining participants were recruited randomly from participants who were found to be negative on the WHO tool (n = 62538). From the screened negative group 1000 participants were approached and 563 (56.3 %) agreed to take part and provided data. From September 2011 to June 2012, using a structured, pretested, interviewer administrated questionnaire participants were interviewed to obtain information on (i) socio-demographic characteristics, (ii) three days dietary intake history including fruits and vegetables intake [consumption were assessed by a question that inquired number of serving (medium portions) of either fruits or any vegetables including green leafy per day], (iii) smoking status including type of smoking and/or smokeless tobacco use, past smoking history (iv) alcohol intake including local form of alcoholic beverages, (v) physical activity assessed by exact daily duration (minutes) of work related, commute related and leisure time related physical activity, and (vi) history of medicine intake for any chronic disease management.

### Clinical measures

For health examination, the parameters included anthropometric and blood pressure measurements. Variables recorded were height (HT), weight (WT), waist circumference (WC), hip circumference (HC) and blood pressure (BP). To ensure data uniformity similar equipment were used by all data collectors. In addition all instruments were calibrated regularly before data collection. The health examination element involved measurement of HT by a portable, locally manufactured, stadiometer, standing upright on a flat surface machine; WT using modern electronic digital LCD weighing machines; WC and HC were measured using spring tapes and following standard procedures [8]. BP was measured in the right arm in both sitting and standing position using India Mart Ambala (Advanced Technocracy Inc, indiamart®, India) instruments. Measurement was done by auscultatory method on the day of interview. Prior to the measurement, 10 min rest was assured. Two readings were taken 5 min apart, and if the two reading varied more than 5 mm of Hg, a third reading was taken and the mean of the three readings was considered as final blood pressure of the individual and final data for data analysis. Hypertension was defined as a systolic blood pressure (SBP) of ≥140 mmHg and diastolic blood pressure (DBP) of ≥90 mmHg [9].

### Biochemical measures

Fasting samples were obtained from all individuals after an overnight fast of at least 8–10 h. With proper aseptic precaution, 8 ml of venous blood sample was collected from each participant to measure fasting blood glucose (FBG), total cholesterol (TC), low-density lipoprotein cholesterol (LDL-C), triglycerides (TG) and high density lipoprotein cholesterol (HDL-C). All participants other than those with known diabetes (n = 5) were then given a 75-g oral glucose solution (75 g of oral glucose in 250 mL of water) to drink. After 2 h, another 3 mL of venous blood was collected to determine 2-h post-oral glucose tolerance test (OGTT). After collecting blood, samples were centrifuged on site within 3 h and plasma samples were then collected and were transferred using ice gel-packed cooling boxes from field to TSH to refrigerated and stored at −70 °C. From TSH, using dry ice, samples were shifted to the laboratory of the Bangladesh Institute of Research and Rehabilitation for Diabetes, Endocrine and Metabolic Disorders (BIRDEM) and stored at −70 °C until laboratory assays were carried out. Plasma glucose was measured by the glucose oxidase method using DimalesionRxL Max (Siemens AG, Erlangen, Germany). Quality control of the blood glucose measurement was checked by measuring the 2-h plasma glucose values in every 20th case. The intra-assay coefficient of variation was 1.08 % at a mean of 5.30 mmol/L, and the inter-assay coefficient of variation was 2.01 % at a mean of 5.39 mmol/L. TC, TG, HDL-C were analysed by enzymatic colorimetric method and LDL-C was estimated by Friedewald’s formula.

### Classification criteria

Smokers included subjects who smoked cigarettes, bidis, or other forms of tobacco daily. Users of other forms of tobacco (mainly chewed tobacco and betel nut habit) were classified as non-smoked tobacco use. Less than 1 servings of fruits or less than 3 serving of vegetables daily were categorized as low dietary intake [10]. Those with no regular work-related or leisure-time physical activity were classified as having physical inactivity [11]. Asian BMI criteria was used to categorise and define underweight (less than 18 · 5 kg/m2), normal (18.5-23.0 kg/m^2^), overweight (23–27.5 kg/m^2^) and obesity (higher than 27.5 kg/m^2^) for both sexes [12]. Abdominal obesity was diagnosed when WHR was >0.90 in men and >0.80 in women or waist circumference was >95 cm in men and >80 cm in women according to the internationally harmonized definition of metabolic syndrome for South Asians [13] and WHtR for both sexes were ≥0.50 [14]. HTN were diagnosed if individual’s average systolic BP was ≥140 mmHg or diastolic BP was ≥90 mmHg, or if they were receiving treatment for HTN [14, 15]. Diabetes mellitus (DM) was defined as FBG ≥7.0 mmol/L and/or 2 h after 75-g oral glucose solution ≥11.1 mmol/L and pre-DM followed by the WHO guideline [16]. In addition, known DM was defined by the use of insulin or oral anti-diabetic medication(s) and self-reported DM. Dyslipidaemia was defined if serum total cholesterol high (≥200 mg/dl), triglycerides high (≥150 mg/dl), LDL cholesterol high (≥130 mg/dl) or HDL cholesterol low (<40 mg/dl in men and <50 mg/dl in women) according to the National Cholesterol Education Program Adult Treatment Panel-3 (NCEP-ATP-3) guidelines [17]. Creatinine was defined high (>1.2 mg/dl). Income was classified according to the 2006 per capita Gross National Income (GNI) and according to World Bank (WB) calculations [18].

Verbal consent in presence of witnesses for all participants was obtained. The NB-NCDP study protocol was approved earlier from Human Research Ethical Committee (HREC) of the University of New South Wales (HREC ref: ≠HC12621), Sydney, Australia and the Ethics Review Committee of the Diabetic Association of Bangladesh (BADAS).

### Statistical analysis

Percentages were used to describe the prevalence rates of risk factors for CVDs. Means and standard deviations (SD) were used for continuous variable and to summarize categorical data both the number and proportion for all the socio-demographic, behavioural, anthropometric, clinical and biochemical parameters of the study were used. Independent-sample *t*-tests (for continuous variables) and Pearson’s chi-square test (for categorical variable) were done as to perform intergroup comparison to examine the difference in the covariates between high risk and not high risk participants.

We examined the associations between socio-demographic, behavioural, anthropometric, clinical and biochemical measures among high risk and not high risk participants. To decide the final model of association for each individual with CVDs, a backward elimination approach of model building was used. We first fitted univariable logistic regression with all the predictors. Logistic regression models were also fitted with interaction terms between pairs of explanatory variables (e.g., age and gender, etc.). Variables those were significant at 15 % level in the univariable logistic regression models were included in a multivariable base model. For interaction terms, the criterion for significance to include in the base model was 5 % level. None of the interaction terms were significant and so was not included in the base model. From the base model, variables were excluded one by one based on their P-values (P > 0.05) to reach the final model. Following the development of final model with the backward elimination method, we checked for multi-collinearity by estimating a variance inflation factor (VIF) by fitting a multiple linear regression model with the binary outcome variable [19]. The model building was conducted manually, not using an automatic variable selection method. Data were analysed using Stata version 12.

## Results

Overall social and demographic characteristic of all the 1170 high risk participants and 563 not high risk participants are shown in the Table 1. High risk individuals differed from the not high risk participants with respect to gender and employment status (*p* < 0.001). Participants aged between 31–45 and 46–60 years comprised the largest number of high risk (80 %) participants compared to other age groups. Females were over-represented (60 %) in the high risk group. About three quarters of the participants (73 %) had low or poor educational knowledge (i.e., illiterate/only signature/gonoshikha and primary education) and almost 99 % were low and lower middle income group participants. Among high risk and not high risk group; 72.3 % and 54.4 % of the study participants respectively were from physically active group (i.e., house maker/farmer).

**Table 1:** Socio demographic characteristics of the study participants (n = 1733)

Variables^a^		High risk (n = 1170)	Not high risk (n = 563)	*P* value^*^
Gender				
	Male	463 (39.6)	372 (66.1)	<0.001
	Female	707 (60.4)	191 (33.9)	
Age (years) (M ± SD)		50.27 ± 10.83	50.46 ± 10.86	0.777
	31- 45^b^	459 (39.2)	212 (37.7)	0.737
	46- 60	485 (41.5)	244 (43.3)	
	61 year& above	226 (19.3)	107 (19.0)	
Religion				
	Islam	796 (68.0)	377 (67.0)	0.386
	Hindu	358 (30.6)	180 (32.0)	
	Christian	16 (1.4)	6 (1.1)	
Family size				
	Small (less than 4 persons)	564 (48.2)	274 (48.7)	0.275
	Medium (5 – 8 persons)	541 (46.2)	273 (48.5)	
	Large (>9)	65 (5.6)	16 (2.8)	
Type of family				
	Nuclear family	982 (83.9)	475 (84.4)	0.815
	Joint family	188 (16.1)	88 (15.6)	
Marital status				
	Never married/Divorced/ Separated/ Widowed	129 (11.5)	66 (11.7)	0.887
	Married	1037 (88.6)	497 (88.3)	
Education				
	Illiterate/ Signature/Gonoshikha	512 (43.8)	249 (44.2)	0.729
	Primary level	336 (28.7)	163 (29.0)	
	Secondary level and above	322 (27.5)	151 (26.8)	
Gross National Income (per capita, US$)				
	Low income (≤905)	345 (29.5)	150 (26.6)	0.175
	Lower-middle income (906–3595)	818 (69.9)	410 (72.8)	
	Upper-middle income (3596–11115)	7 (0.6)	3 (0.5)	
Employment status				
	Unemployed/ sacked from the present job/ Retired	78 (6.7)	25 (4.4)	0.001
	Office work/ Business/ Skilled labour	104 (8.9)	85 (15.1)	
	House maker/farmer	846 (72.3)	306 (54.4)	
	Rickshaw puller/day labour/ Others	142 (12.1)	147 (26.1)	

*SD*, standard deviation; *yrs*, years

^*^For continuous variables *p*-values were obtained by doing independent samples *t*-test and for categorical variable from chi-squared test

^a^Values expressed as numbers and percentages in parentheses or mean ± SD, as appropriate; ^b^age group applies as to baseline cohort in 2008

Prevalence of behavioural, anthropometric and other risk factors is shown in Table 2. Among all participants, rate of current smokers, past smokers and smoking 100 sticks in life time were 23.6 %, 22.6 % and 30.6 % respectively. Moreover regular betel nut chewing, occasionally and leaf format tobacco along with betel nut were 36.6 %, 18.5 % and 19.0 % respectively. Prevalence of overweight and obesity in total as BMI were 39.1 % and 20.5 % in high risk and not high risk participants respectively Except for physical activity and smokeless tobacco consumption all the anthropometric, behavioural and other risk factors were significantly higher among high risk group compared to not high risk individuals (*p* < 0.001).

**Table 2:** Behavioral and anthropometric risk factors among high risk and not high risk study participants

Variables^a^	High risk (n = 1170)	Not high risk (n = 563)	*P* value^*^
Smoking Pattern			
Non-smoker	836 (71.5)	278 (49.4)	0.001
Smoker	334 (28.5)	285 (50.6)	
Smokeless tobacco			
Non smokeless tobacco	543 (46.4)	240 (42.6)	
Regular smokeless tobacco	434 (37.1)	196 (34.8)	0.139
Occasional smokeless tobacco	193 (16.5)	127 (22.6)	
Physical activities pattern (based on PAL)			
Inactive (<1.40)	168 (14.4)	88 (15.6)	
Low active (1.40 to 1.59)	200 (17.1)	97 (17.2)	0.359
Active (1.6 to 1.89)	165 (14.1)	88 (15.6)	
Very active (>1.90)	634 (54.3)	290 (51.5)	
Fruits intake pattern			
Less than 1 servings/day	1163 (99.4)	458 (81.3)	<0.001
1-2 servings/day	7 (0.6)	105 (18.7)	
Vegetables intake pattern			
Less than 2 servings/day	487 (41.7)	324 (57.8)	<0.001
3-5 servings/day	681 (58.3)	237 (42.2)	
BMI (M ± SD)	22.24 ± 4.40	20.35 ± 3.28	<0.001
Normal (18.51-23.0)	524 (44.8)	276 (49.0)	
Underweight (<18.5)	188 (16.1)	170 (30.2)	0.001
Overweight (23.01-27.5)	348 (29.8)	100 (17.8)	
Obese (>27.01)	109 (9.3)	15 (2.7)	
Waist circumference	79.53 ± 13.18	74.92 ± 9.86	<0.001
Normal (<0.90 male, <0.80 female)	755 (64.5)	496 (88.1)	0.001
High risk (>0.90 male, >0.80 female)	415 (35.5)	67 (11.9)	
Waist Hip Ratio	0.91 ± 0.11	0.90 ± 0.10	0.134
Normal (<0.95 male, <0.80 female)	313 (27.1)	305 (54.2)	
Moderate (0.96-1.0 male, 0.81-0.85 female)	223 (19.1)	103 (18.3)	<0.001
High risk (>1.0 male, >0.85 female)	617 (53.5)	148 (26.3)	
Waist Height Ratio	0.51 ± 0.09	0.47 ± 0.06	0.001
<=0.5(non central fat distribution - pears)	513 (43.8)	403 (71.6)	<0.001
>0.5(central fat distribution - apples)	657 (56.2)	160 (28.4)	

^*^For continuous variables *p*-values were obtained by doing independent samples *t*-test and for categorical variable from chi-squared test

^a^Values expressed as numbers and percentages in parentheses

Clinical and biochemical risk factors prevalence is shown in Table 3. In high risk and not high risk participants respectively: prevalence of HTN was 16.6 % and 3.7 %; pre HTN was 41.7 % and 11.9 %; pre-diabetes was 27.7 % and 21.7 %; all the lipid profile markers (i.e., serum cholesterol, TG, HDL and LDL) were high in an average 30 % for all participants and overall dyslipidaemia were 85.8 % and 89.5 %. The prevalence of self-reported DM among study participants was very low (only 5 individuals). With the exception of LDL and creatinine; all the clinical and biochemical risk markers were significantly higher among high risk compared with not high risk individuals (*p* < 0.001 and *p* < 0.05). Among all the study participants, 78.7 % had presence of at least two or more known risk (i.e., DM, HTN, dyslipidaemia or obesity), among them 91 % were female and 64.7 % were male.

**Table 3:** Clinical and biochemical risk factors among high risk and not high risk study participants

Variables^a^	High risk (n = 1170)	Not high risk (n = 563)	*P* value^*^
Systolic blood pressure (mmHg)	138 ± 21	117 ± 17	<0.001
Normal (≤140 mmHg)	737 (64.9)	512 (90.9)	<0.001
High (≥140 mmHg)	399 (35.1)	51 (9.1)	
Diastolic blood pressure (mmHg)	86 ± 11	75 ± 10	<0.001
Normal (≤90 mmHg)	885 (77.9)	536 (95.2)	<0.001
High (≥90 mmHg)	251 (22.1)	27 (4.8)	
Hypertension			
Normotensive	473 (40.4)	475 (84.4)	
Pre-hypertensive	512 (43.8)	68 (12.1)	0.001
Hypertensive	185 (15.8)	20 (3.6)	
Fasting blood glucose (mmol/l)	5.41 ± 1.76	5.12 ± 1.55	<0.001
2 h after 75gm glucose (mmol/l)	6.78 ± 3.37	5.79 ± 2.60	<0.001
Glycemic Status			
Non diabetic	808 (69.1)	412 (73.2)	
Pre-diabetic	198 (16.9)	68 (12.1)	0.015
Diabetic	164 (14.0)	83 (14.7)	
Cholesterol (mg/dl)	186 ± 42	180 ± 56	0.040
<200 normal	853 (74.3)	458 (81.8)	
200.01 - 240 border line high	206 (17.9)	80 (14.3)	<0.001
>240.01 high	89 (7.8)	22 (3.9)	
Triglyceride (mg/dl)	152 ± 77	140 ± 71	0.002
<150 normal	705 (61.5)	388 (69.3)	
150.01-200 border line high	231 (20.2)	105 (18.8)	<0.001
>200.01 high	210 (18.3)	67 (12.0)	
HDL (mg/dl)	41 ± 8	40 ± 8	0.044
Normal (male >40, Female >50)	333 (28.5)	207 (36.8)	0.001
Risk (male < 40, Female < 50)	837 (71.5)	356 (63.2)	
LDL (mg/dl)	114 ± 38	111 ± 53	0.198
Normal (LDL < 100)	417 (35.6)	225 (40.0)	
Near normal (LDL ≥ 100.01 & < 130)	412 (35.2)	178 (31.6)	0.844
High (LDL ≥ 130.01 & < 190)	266 (22.7)	101 (17.9)	
Very high (LDL > 190.01)	75 (6.4)	59 (10.5)	
Dyslipidaemia			
No	163 (14.2)	59 (10.5)	0.026
Yes	983 (85.8)	501 (89.5)	
Creatinine (mg/dl)	0.97 ± 0.16	0.99 ± 0.12	0.002
Normal	1085 (97.0)	515 (98.1)	0.181
Abnormal	33 (3.0)	10 (1.9)	

^*^For continuous variables *p*-values were obtained by doing independent samples *t*-test and for categorical variable from chi-squared test

^a^Values expressed as numbers and percentages in parentheses

In a multivariable logistic regression model the variable gender, fruits and vegetable intake pattern, smoking, BMI, WHR, WHtR, HTN and dyslipidemia were significantly associated to the participants CVD risk status (high risk/ not high risk) after adjusting for other variables.

## Discussion

The study found a high prevalence of CVD risk factors even in a relatively traditional rural^1^ population in Bangladesh. A notable finding was that the proportion of females in the high risk group was almost twice the proportion in the low risk group. On multivariate analysis this nearly twofold association of female sex with high CVD risk status (OR 1.87, 95%ci 1.34-2.59) remained present. Usually males are reported to be more susceptible to CVDs [20] and thus the present finding of female predominance in this population needs further in-depth investigation.

A larger proportion of the high risk subjects in the present study are from lower socioeconomic and educational levels and they are homemakers and farmers. Again, this is in contrast to the usual notion that CVDs are more common among higher socioeconomic classes [11]. A majority of these people are active or very active and thus, unlike other studies on urban or urbanizing rural populations in Bangladesh [21–23], inactivity does not seem to be a major determinant of CVD high risk in this population.

Smoking is a well-known risk factor of CVDs [24] and high rates of smoking both in urban (42.3 %) [25] and rural (36.1 %) [26] populations in Bangladesh have been previously reported. In the present study even a higher proportion of smokers (50.6 %) has been found among the not high risk participants. There is the seemingly paradoxical finding of relatively lower (28.5 %) proportion of smokers among the high risk groups. However, given the fact that these people were identified (by the WHO Screening Tool) on the basis of cardiovascular symptoms and thus had already undergone some form of treatment, many of them may have quit smoking following medical advice. A similar paradox is seen regarding low fruits and vegetable intake which is known to be another risk factor for CVDs. A large proportion of subjects in both the groups had less than 1 servings/ day of fruits and less than 2 servings/ day of vegetable intake; however, the proportion of low intake was substantially higher for fruits and lower for vegetables in the high risk compared to the not high risk participants respectively (*p* < 0.001, Table 2). Again, advice from the health care providers and, additionally, support from families may have played a role in improving nutritional balance in the high risk group.

A very important finding in the present study is the prevalence of overweight and obesity (both general and central) in such a remote rural population. It is alarming that, irrespective of risk groups, both general (on the basis of BMI) and central (on the basis of WC, WHR and WHtR) obesity are quite high in this population. It is understandable that all these anthropometric measures are significantly more prevalent among the high risk group compared to the not high risk group. The finding was paralleled by the strong association (as revealed by OR) of all these measures with high risk on multivariable analysis. There is no recent study conducted in Bangladesh on a similar peripheral rural population. However, a few studies on populations close to urban areas report a prevalence of overweight as 17.2 % [27] and obesity as 24.4 % [28]. In these studies the rates of central obesity (with WC/WHR/WHtR as indicators) were reported to be 37.9 %, 69.8 % and 58.1 % [28]. There is evidence that abdominal and visceral fat deposits lead to pro-inflammatory profiles, dyslipidaemia, insulin resistance and other metabolic syndrome factors that promote atherosclerosis [29]. Abdominal obesity has also been shown as a risk factor in CVDs by various large scale epidemiological studies [30] mostly in Europe. Moreover, a recent study in Bangladesh has shown that after adjusting BMI, HTN and other confounding variables; WHR and WHtR showed better risk prediction for early CVDs [31]. Therefore in addition to BMI, these WC and WHR measures need to be considered as supplemental indices for re-defining obesity as well as risk factors for CVDs. These increasing trends of obesity indicators suggest that the transition in lifestyle among the rural population of Bangladesh may be rapidly producing adverse changes that could accelerate the CVDs burden.

Hypertension was found to be the single most important risk factor for CVDs in this study. A large proportion of the high risk participants were found to have either hypertension or pre-hypertension (Table 3) and these conditions were found to be associated with the increased CVDs risk by 6.85 and 8.10 times respectively after adjusting for some confounding variables (Table 4). The finding conforms with the reports or recent non-communicable disease surveillance in Bangladesh as well as the INTERHEART study [32, 33].

**Table 4:** Multivariable analyses of high risk and not high study participants (n = 1733) by the following characteristics

Variables		Multivariable analysis	
		OR^a^(95 % CI)	*P* value
Age (years)	31 – 45	1.00	
	46 - 60	0.85 (0.65-1.12)	0.256
	Above 60 year	0.92 (0.64-1.31)	0.637
Gender	Male	1.00	
	Female	1.87 (1.34-2.59)	<0.001
Economic status	Low income	1.00	
	Lower- middle income	0.93 (0.71-1.22)	0.604
	Upper- middle income	1.15 (0.19-6.91)	0.878
Employment status	Unemployed/ sacked from the present job/ Retired	1.00	
	Office work/ Business/ Skilled labour	0.70 (0.37-1.32)	0.269
	House maker/farmer	0.91 (0.52-1.59)	0.729
	Rickshaw puller/day labour/ Others	0.64 (0.35-1.17)	0.151
Fruits intake pattern	More than 1 servings/day	1.00	<0.001
	Less than 1 servings/day	50.50 (21.56-118.29)	
Vegetable intake pattern	less than 2 servings/day	1.00	
	3-5 servings/day	0.70 (0.54-0.91)	0.007
Smoking	No	1.00	
	Yes	0.65 (0.49-0.87)	0.004
Smokeless tobacco	No	1.00	
	Yes	1.28 (1.00-1.63)	0.052
BMI^b^	Normal (18.51-23.0)	1.00	
	Underweight (<18.5)	0.62 (0.46-0.84)	0.002
	Overweight (23.01-25.0)	1.65 (1.22-2.25)	0.001
	Obese (>25.01)	2.40 (1.29-4.49)	0.006
WC^b^	Normal (<0.90 male, <0.80 female)	1.00	
	Risk (>0.90 male, >0.80 female)	2.65 (1.92-3.67)	<0.001
WHR^b^	Normal (<0.95 male, <0.80 female)	1.00	
	Moderate (0.96-1.0 male, 0.81-0.85 female)	1.56 (1.09-2.22)	0.014
	High risk (>1.0 male, >0.85 female)	2.49 (1.74-3.56)	<0.001
WHtR^b^	<=0.5(non central fat distribution - pears)	1.00	
	>0.5(central fat distribution - apples)	2.48 (1.93-3.20)	<0.001
Hypertension	Normotensive	1.00	
	Pre-hypertensive	6.85 (5.02-9.34)	<0.001
	Hypertensive	8.10 (4.97-13.21)	<0.001
Glycaemic status	Non diabetic	1.00	
	Pre-diabetic	0.96 (0.57-1.61)	0.869
	Diabetic	1.06 (0.80-1.41)	0.679
Dyslipidaemia	No	1.00	
	Yes	0.63 (0.43-0.91)	0.013
Creatinine (mg/dl)	Normal	1.00	
	Abnormal	1.79 (0.75-4.27)	0.187

^a^Adjusted odds ratio after multivariable logistic regression; ^b^Different obesity indicators (i.e., BMI, WC, WHR and WHtR were added in the model separately)

The proportion of diabetes and pre-diabetes is reasonably high in both the not high risk and high risk groups. For prevalence, although the two groups vary significantly, the differences are still not large and this is reflected in the lack of effect of glycemic status on CVD risk on multivariate analysis. The blood glucose values, both in high and not high risk participants (Table 3), are not particularly high and thus most of the diabetic subjects are probably in good control. This, in turn, may be due to the relatively organized diabetes care network in Bangladesh. Thus the effects of diabetes may have been offset by good control, particularly in the high risk participants.

A surprisingly high proportion of both high and not high risk participants were found to have dyslipidemia with at least on abnormal component of lipid profile (Total cholesterol, TG, HDL and LDL). The risk was mostly contributed by low HDL, borderline or high TG, high or very high LDL, and borderline or high cholesterol. Again, due to a scarcity of recent studies in similar settings it is difficult to compare previous data with the present study. Given the low intake of fat in this population the high prevalence of dyslipidemia needs further investigation.

Since there are no existing studies on such a remote rural population in Bangladesh it is difficult to directly compare the present data with previously generated data on Bangladeshi population. Several studies in Bangladesh from rural populations reported a prevalence of HTN 6.6 % - 19.1 % [27, 34–39], Pre DM and DM 8.6 % - 22.4 % and 1.7 % - 8.2 % [28, 35, 37, 40–42], dyslipidemia 4.8 % - 28.7 % and overweight/obesity 7.8 % - 24.4 % [28, 43–45]. The corresponding values for the urban population in Bangladesh were HTN 14.7 % - 44.80 % [23, 46–48], DM 6.1 % - 35.3 % [40, 45, 48] dyslipidemia 0.1 % - 64.0 % and overweight/obesity 20.9 % - 63.1 % [45, 48]. Our findings are consistent with the ranges provided in the aforementioned studies. The wide ranges in prevalence might occur due to variations in study quality, age group, selection of participants, risk factors diagnosis (self-reported and through measurement, measurement instruments etc.).

### Study strengths and limitations

The strengths of the present study included large sample size and population-based design. However, the main weakness of this study is using the WHO recommended 2002 tool as it only identifies a subset of the high CVD risk participants (i.e. those who are at high risk because of existing CVD). It does not detect those who would be at high risk if all the relevant clinical and biochemical measures could be performed (BP, lipid profile, glucose abnormality) to calculate absolute risk. As we used this WHO tool during the 2011–2012 screening, it was to some extent focused on a clinical measure of cardiovascular disease. Therefore it is a possibility of overestimation of the prevalence of hypertensive disorders in the high risk participants when the average general population is considered. Also there is a possibility of differences in recall and reporting between men and women, with women perhaps being more likely to report symptoms. On the other hand, the exclusion of the persons with high propensity for subclinical CVDs may lead to some underestimation of the burden among the not high participants. Thus, it seems that the true prevalence of pre-hypertension and hypertension among adults in this population lies somewhere in between 16 % to 59.6 % and this needed to be explored through further studies with representative sampling. Similar works are required to estimate the burden of obesity and overweight (between 20 - 40 %) in this population. In contrast to these two risk factors the prevalence of dysglycemia and dyslipidemia do not differ significantly between the high and not high risk study participants and thus, the values may be considered to represent those of the general population.

Moreover our sample of CVDs may not be representative of all parts of Bangladesh as it is one of the remote areas in the North where people survive with a below average GDP rate. However, the demographic characteristics of the study area were similar with respect to sex ratio, per-capita income, household size, literacy rate, life expectancy, occupation, and marital status to those in other rural areas of Bangladesh and similar as national average [49], and the study proved helpful in understanding CVDs risk score and CVD risk factors prevalent among the rural population. A random measurement error would bias the association towards null, particularly for smoking, physical activity and for dietary intake; these biases could influence the overall study findings. However, we used an average of three day dietary recall methods to minimize the recall bias. We also used yearly physical activity history on parallel with weekly pattern, to control and minimize random measurement error. The recall bias would be non-differential and might have further pooled the association towards null. Lastly, we did not include family history of CVDs in our detailed questionnaire. It is possible that some other risk factors that play a role in CVDs in the rural environment are yet to be explored.

## Conclusion

In conclusion, the present study showed that the prevalence of clinical and biochemical risk factors of CVDs are quite high, even in a remote rural Bangladeshi population. This may be related to the socioeconomic and cultural transition in Bangladeshi society. Surprisingly, more of the high risk group was female and there were fewer smokers. Obesity and hypertension were more frequent in high risk participants. In particular, central obesity is common despite the high level of physical activity. Public health strategies to address overweight and obesity are needed. The data also shows that the most important biochemical risk factors of CVDs are not addressed by the WHO CVD Risk Screening Tool for Low – and Medium-Resource settings.

## Footnotes

1 Most of the existing research conducted in Bangladeshi rural settings has been in rural areas adjacent to urban spaces, whereas a traditional rural area is one geographically and behaviorally distinct from urban influences.
